# Supplementary material for: Neurobiological Signatures of Auditory False Perception and Phantom Perception as a Consequence of Sensory Prediction Errors
Source: Biology (Basel). 2022 Oct 13;11(10):1501. doi: 10.3390/biology11101501 (PMC9598671; doi:10.3390/biology11101501)

Supplementary Figure S1. Functional connectivity in healthy subjects during an auditory prestimulus time window (-300 ms to 0 ms) in the Talk and Listen paradigms

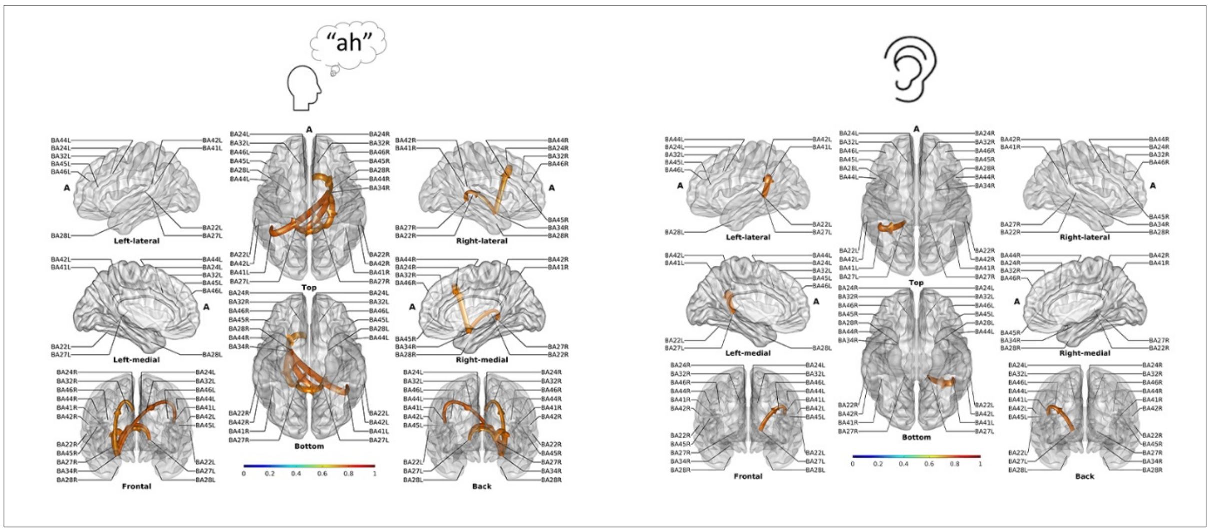

Supplementary Figure S2. Functional connectivity in healthy subjects during a stimulus time window (0 to 200 ms) in the Talk and Listen paradigms

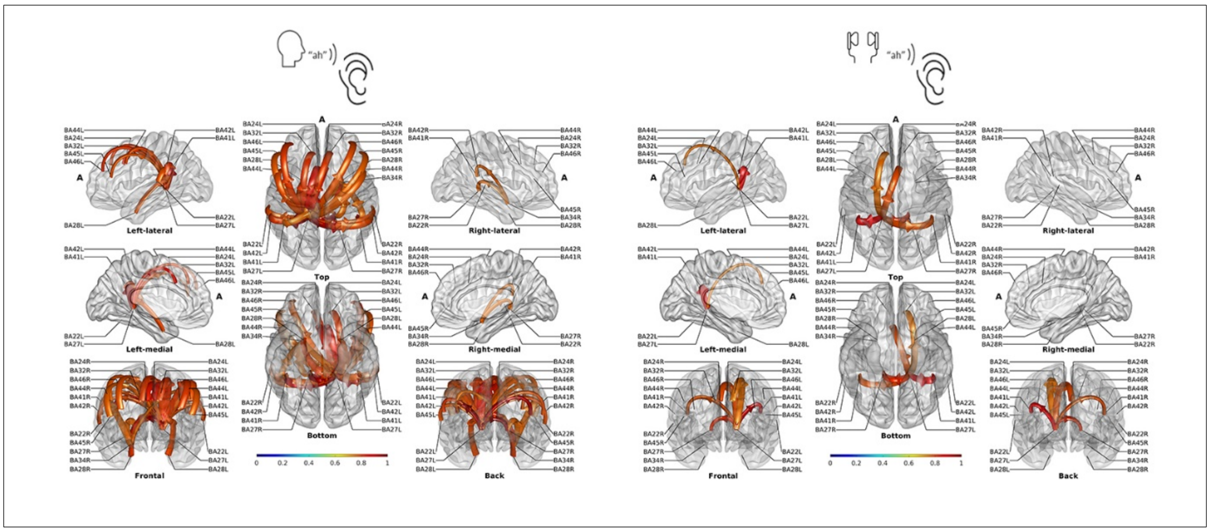

Supplementary Figure S3. Functional connectivity in schizophrenia during a stimulus time window (0 to 200 ms) in the Talk and Listen paradigm

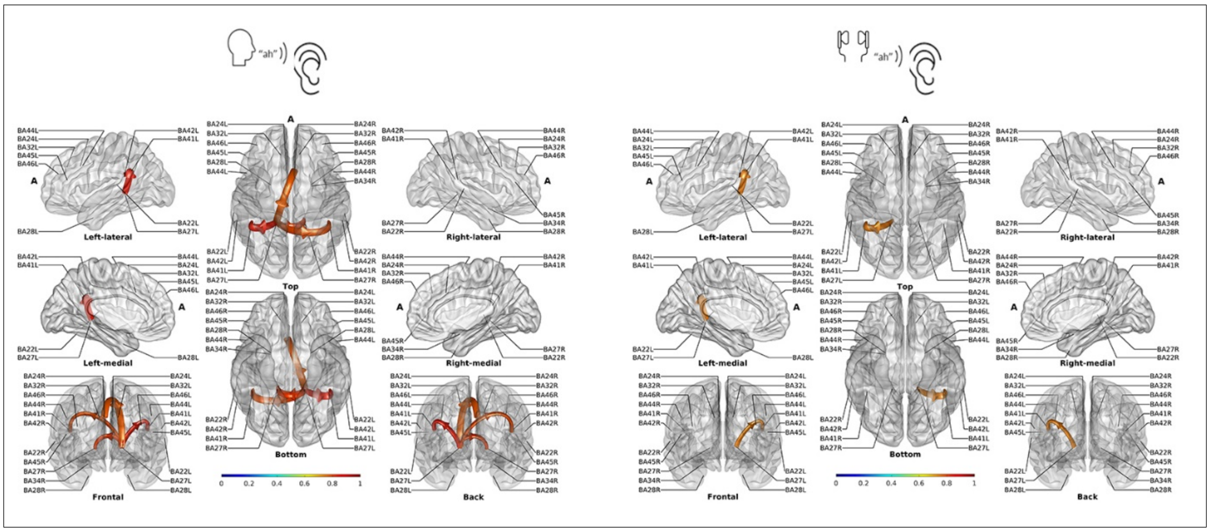

Supplementary Figure S4. Granger causal connectivity of grand-averaged evoked alpha activity during an auditory prestimulus time window before the exclusion of BA34L (a) and after the exclusion of BA 34L (b)

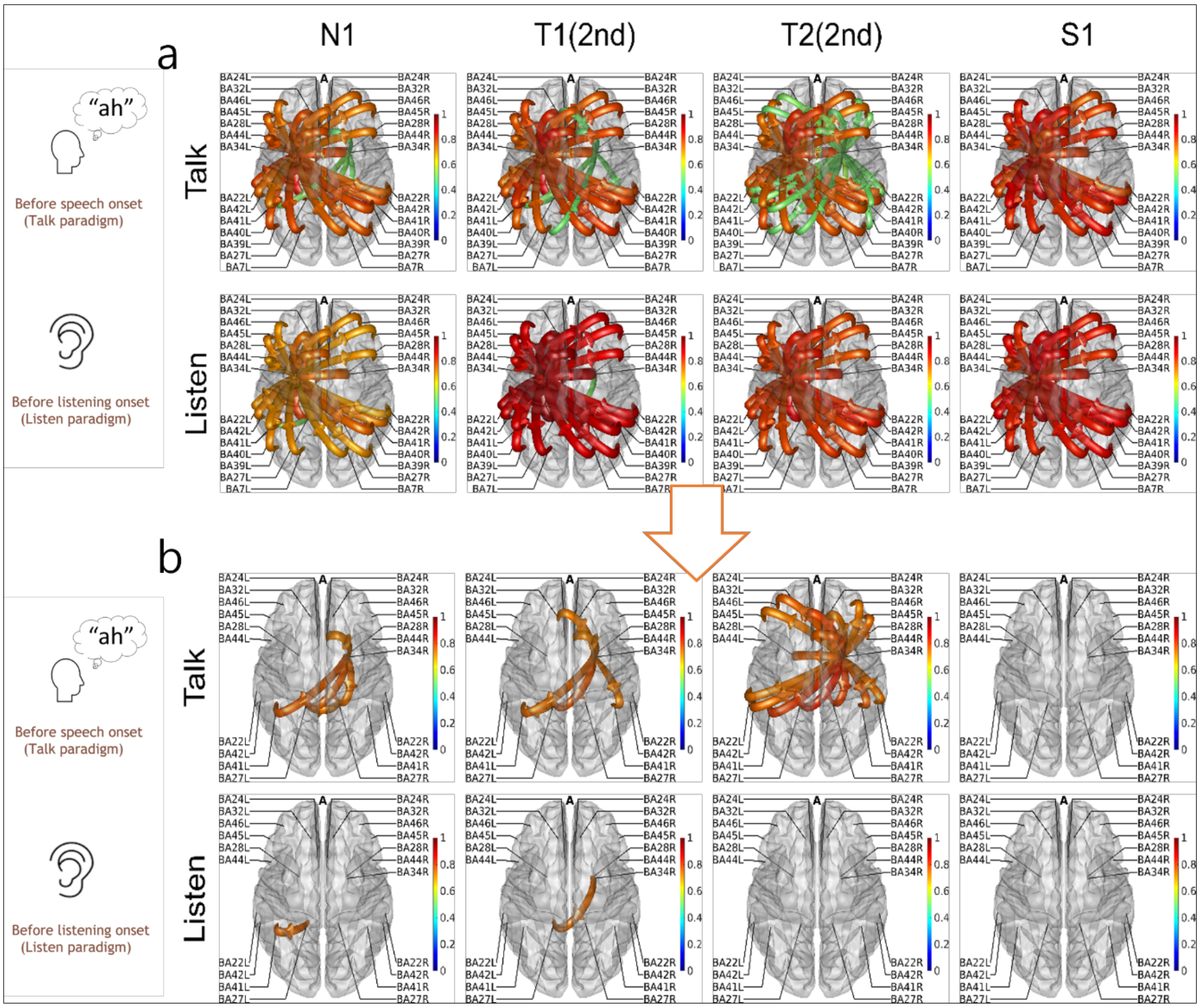

Supplement: Supplementary file 1 [file biology-11-01501-s001.zip › biology-1938405-supplementary.pdf]
